# Association of Blood Pressure at Successful Recanalization and Parenchymal Hemorrhage After Mechanical Thrombectomy With General Anesthesia

**DOI:** 10.3389/fneur.2020.582639

**Published:** 2020-11-17

**Authors:** Hui Cheng, Chao Xu, Xing Jin, Yigang Chen, Xu Zheng, Feina Shi, Xudong He, Yonggang Hao, Yun Jiang, Jinhua Zhang, Zhicai Chen

**Affiliations:** ^1^Department of Neurology, Sir Run Run Shaw Hospital, School of Medicine, Zhejiang University, Hangzhou, China; ^2^Department of Neurology, Zhejiang Provincial People's Hospital, Hangzhou, China; ^3^Department of Neurology, The Second Affiliated Hospital, School of Medicine, Zhejiang University, Hangzhou, China

**Keywords:** blood pressure, mechanical thrombectomy, hemorrhagic transformation, recanalization, large artery occlusion

## Abstract

**Background:** This study aims to investigate the association between blood pressure (BP) at the time of recanalization and hemorrhagic transformation in large vessel occlusion (LVO) patients following mechanical thrombectomy (MT) with general anesthesia.

**Methods:** We retrospectively reviewed our data base for patients with acute ischemic stroke acute ischemic stroke (AIS) who received MT between January 2018 and December 2019. The BP at two adjacent time points immediately after successful recanalization was recorded for subsequent calculation of mean BP (BP_mean_), maximum BP (BP_max_), minimum BP (BP_min_), range of BP (BP_range_), and standard deviation of SP (BP_SD_). Hemorrhagic transformation was identified on 24-h computerized tomography images according to the European Cooperative Acute Stroke Study (ECASS) III trial. We used binary logistic regression analysis to investigate the association of BP parameters and the incidence of parenchymal hemorrhage (PH) and PH-2.

**Results:** A total of 124 patients with anterior circulation LVO were finally included for analyses. After controlling for intravenous thrombolysis, procedure duration of mechanical thrombectomy, baseline National institutes of Health Stroke Scale (NIHSS), baseline ASPECTS, and number of device passes, the results showed that every increment of 10 mmHg in SBP_range_ (OR 1.559; 95% CI 1.027–2.365; *P* = 0.037) and SBP_SD_ (OR 1.998; 95% CI 1.017–3.925; *P* = 0.045) were independently associated with PH. After adjustment for intravenous thrombolysis, procedure duration of mechanical thrombectomy, baseline NIHSS, the results showed that every increment of 10 mmHg in SBP_mean_ (OR 1.973; 95% CI 1.190–3.271; *P* = 0.008), SBP_max_ (OR 1.838; 95% CI 1.199 to 2.815; *P* = 0.005), SBP_range_ (OR 1.908; 95% CI 1.161–3.136; *P* = 0.011) and SBP_SD_ (OR 2.573; 95% CI 1.170–5.675; *P* = 0.019) were independently associated with PH-2.

**Conclusion:** Patients with higher systolic BP and variability at the time of successful recanalization were more likely to have PH-2 in LVO patients following MT with general anesthesia.

## Introduction

Mechanical thrombectomy (MT) for large vessel occlusion (LVO) has proved to be the new standard of therapy in acute ischemic stroke (AIS) (1). But more than 50% of patients still have unfavorable outcomes after early successful recanalization (2). The most severe complication is hemorrhagic transformation (HT), especially parenchymal hemorrhage (PH), which could result in early neurological deterioration and long-term outcomes (3). The current guidelines from the American Heart Association/American Stroke Association guidelines arbitrarily recommend blood pressure (BP) control of <180/105 mm Hg during and after MT. However, data regarding guidance for optimal BP management among patients treated with MT remain scarce (4). Theoretically, the target of BP control should be lower in patients following MT because of the high hemorrhagic transformation risk after clot removal (5).

Several studies have shown that blood pressure after MT is related to hemorrhagic transformation. Goyal et al. have found that elevated maximum systolic BP levels during the first 24 h following MT are independently correlated with worse functional outcomes in LVO patients (6). Another previous study involving 182 patients found that increased BP variability during the first 24 h predicts worse neurologic outcomes in AIS patients treated with intra-arterial therapies (7). In most of the previous studies on the relationship between BP and hemorrhagic transformation, blood pressure was taken after admission to the neurologic intensive care unit. There are few studies on BP at the time of recanalization and hemorrhagic transformation in LVO patients following MT with general anesthesia.

In light of these considerations, we aimed to investigate the relationship between BP at the time of recanalization and hemorrhagic transformation and hypothesized that patients with elevated BP had higher risk of hemorrhagic transformation.

## Methods and Materials

### Study Subjects

MT under general anesthesia is our center's first choice standard procedure. Only in a few cases when the anesthesiologist could not arrive at the angio-suite in time is MT under conscious sedation chosen. Considering the difference of BP levels between patients under general anesthesia and conscious sedation, we excluded patients with conscious sedation in order to reduce the heterogeneity of study subjects. Propofol, sufentanil, and rocuronium were used to induce general anesthesia. Propofol was used for the maintenance of anesthesia. The BP control target in our center is to maintain BP at ≤ 180/105 mmHg during the MT procedure in patients who undergo mechanical thrombectomy. During the operation, BP can be regulated by using vasoactive drugs.

We retrospectively reviewed our data base for LVO patients who received MT from January 2018 to December 2019. This study included patients who (1) had internal carotid artery or middle cerebral artery occlusion, (2) received MT with general anesthesia, (3) achieved thrombolysis in myocardial infarction (TICI) 2b/3 recanalization after the procedure, and (4) had 24–36 h follow-up CT scan for evaluation of hemorrhagic transformation. Patients who had baseline systolic BP (SBP) ≥200 mmHg were excluded before the initial inclusion stage.

We retrieved data including age, gender, baseline National Institutes of Health Stroke Scale (NIHSS) score, baseline systolic BP (SBP), and diastolic BP (DBP) levels, vascular risk factors including atrial fibrillation, diabetes mellitus, hypertension, hyperlipidemia, smoking, and history of stroke/transient ischemic attack (TIA), time from onset to successful recanalization—which was defined by TICI scores of 2b or 3 after M (8) number of devices passes. Hemorrhagic transformation was identified as hemorrhagic infarction (HI) and parenchymal hemorrhage (PH) on 24–36 h CT images according to the European Cooperative Acute Stroke Study (ECASS) III trial. Hematoma occupying <30% of infarcted tissue and having no substantive mass effect was defined as PH-1, and hematoma occupying >30% or more of the infarcted tissue with obvious mass effect was defined as PH-2 (9). The etiologies of stroke were determined according to the Trial of Org 10172 in Acute Stroke Treatment (TOAST) (10).

### Assessment of BP Parameters

Continuous arterial BP during the mechanical thrombectomy was automatically monitored by invasive BP monitoring using an arterial catheter in patients under general anesthesia. BP values were recorded every 15 min. Blood pressure at two adjacent time points immediately after successful recanalization was recorded for subsequent calculation. The maximum (max), minimum (min), average (mean), range (maximum–minimum), and standard deviation (SD) values of BP at the time of successful recanalization were calculated.

### Statistical Analysis

Quantitative variables were presented as mean ± SD or median (interquartile range)—depending on the normality of the distribution—and categorical variables were presented as frequency (percentage). We use Fisher's exact test for dichotomous variables, an independent sample 2-tailed *t*-test, or Mann-Whitney *U*-test for continuous variables. Associations of each BP parameter with PH was determined using binary logistic regression models adjusted by baseline characteristics with a *P* < 0.1 in univariate analyses and some well-recognized confounders, respectively. The receiver operating characteristic (ROC) derived optimal cutoff was determined at the maximal Youden's Index. All statistical analyses were performed using SPSS, Version 23.0 (IBM, Armonk, New York). A *P* < 0.05 was considered statistically significant.

## Results

**Figure 1:**
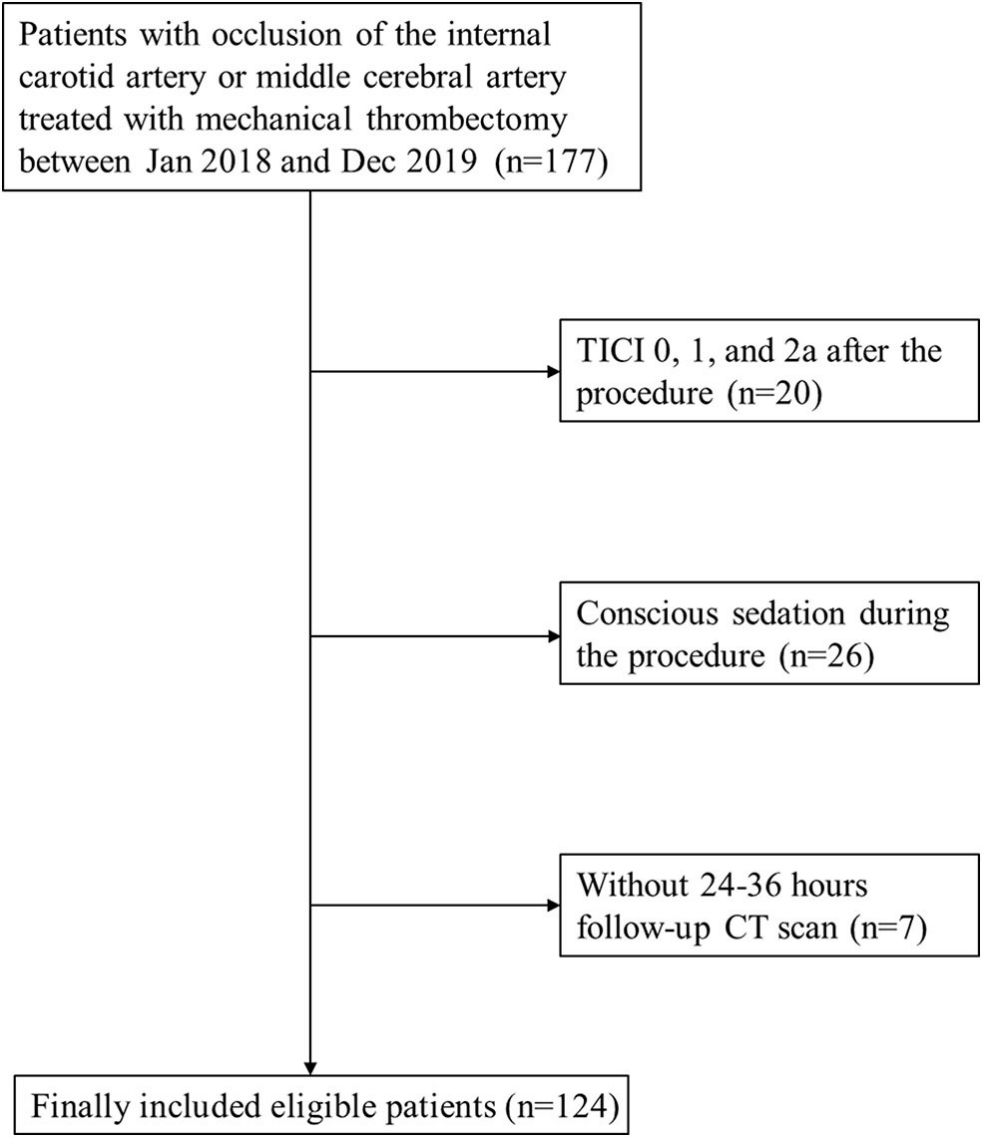
Patient flow chart.

Initially, 177 patients with occlusion of the internal carotid artery or middle cerebral artery treated with MT were included. As shown in Figure 1, a total of 124 patients were finally enrolled after excluding patients due to TICI 0-2a after the procedure (*n* = 20), conscious sedation (*n* = 26), and no 24–36 h follow-up CT scan (*n* = 7).

Of the 124 analyzed patients, the mean age was 66 ± 12 years, and the median baseline NIHSS score was 15 (IQR 11–18) For this group, mean onset-to-reperfusion time was 415 (IQR 317–540) min. A total of 18 (14.5%) had PH at 24–36 h, and 10 of them were PH-2 (8.1%).

### Associations of BP Parameters and Hemorrhagic Transformation

As shown in Table 1, patients with PH had higher baseline NIHSS scores (17 vs. 14, *P* = 0.009), a higher number of device passes, smaller baseline ASPECTS (6 vs. 8, *P* = 0.023), higher 24 h NIHSS (22 vs. 10.5, *P* < 0.001), and lower rate of 3 months mRS ≤ 2 (0 vs. 49, *P* < 0.001) than those without PH. The SBP_max_ (*P* = 0.046), SBP_range_ (*P* = 0.037), and SBP_SD_ (*P* = 0.037) were higher in the PH group. There were no significant differences in other variables, including baseline BP. After controlling for intravenous thrombolysis, procedure duration of mechanical thrombectomy, baseline NIHSS, baseline ASPECTS, and the number of device passes, the results showed that every increment of 10 mmHg in SBP_range_ (OR 1.559; 95% CI 1.027–2.365; *P* = 0.037) and SBP_SD_ (OR 1.998; 95% CI 1.017–3.925; *P* = 0.045) were independently associated with PH (Table 2).

**Table 1:** Univariate analyses of baseline characteristics.

	**PH**		***P*-value**	**PH-2**		***P*-value**
	**Yes (*n* = 18)**	**No (*n* = 106)**		**Yes (*n* = 10)**	**No (*n* = 114)**	
Age (years)	67.7 ± 13.1	65.5 ± 11.4	0.455	71.0 ± 12.2	65.4 ± 11.6	0.149
Male, *n* (%)	11 (61.1)	61 (57.5)	0.777	5 (50.0)	67 (58.8)	0.590
**Risk factors**						
Smoking, *n* (%)	3 (16.7)	25 (23.6)	0.516	2 (20.0)	26 (22.8)	0.839
Hypertension, *n* (%)	10 (55.6)	70 (66.0)	0.390	8 (80.0)	72 (63.2)	0.286
Diabetes mellitus, *n* (%)	3 (16.7)	17 (16.0)	0.947	2 (20.0)	18 (15.8)	0.729
Atrial fibrillation, *n* (%)	10 (55.6)	50 (47.1)	0.510	6 (60.0)	54 (47.4)	0.443
Hyperlipidaemia, *n* (%)	0 (0.0)	2 (1.9)	0.557	0 (0.0)	2 (1.8)	0.673
History of stroke/TIA, *n* (%)	3 (16.7)	17 (16.0)	0.947	2 (20.0)	18 (15.8)	0.729
**Clinical variables**						
Baseline NIHSS (IQR)	17 (13–19)	14 (10–17)	0.009	18 (16–20)	14 (11–17)	0.005
Onset-to-reperfusion time, min	488.0 ± 202.2	454.2 ± 203.7	0.517	445.4 ± 203.3	460.3 ± 203.8	0.824
Baseline SBP, mm Hg	143.1 ± 19.9	143.6 ± 23.2	0.932	140.8 ± 17.1	143.8 ± 23.1	0.687
Baseline DBP, mm Hg	87.1 ± 14.4	84.2 ± 15.9	0.474	87.2 ± 16.3	84.4 ± 15.7	0.602
Bridging thrombolysis, *n* (%)	7 (38.9)	40 (37.7)	0.926	5 (50.0)	42 (36.8)	0.411
Baseline ASPECTS (IQR)	6 (4–8)	8 (6–9)	0.023	7 (4–9)	8 (6–9)	0.537
TOAST classification			0.476			0.739
Cardioembolism, *n* (%)	10 (55.6)	55 (51.9)		6 (60.0)	59 (51.8)	
Large arterial atherosclerosis, *n* (%)	2 (11.1)	26 (24.5)		1 (10.0)	27 (23.7)	
Undetermined Etiology, *n* (%)	6 (33.3)	23 (21.7)		3 (30.0)	26 (22.8)	
Others, *n* (%)	0 (0.0)	2 (1.9)		0 (0.0)	2 (1.8)	
Number of device passes	3 (2–4)	2 (1–3)	0.017	2 (1–5)	2 (1–3)	0.170
Recanalization, *n* (%)	15 (83.3)	75 (70.8)	0.269	9 (90.0)	81 (71.1)	0.198
Procedure duration, min	87.5 ± 46.0	73.8 ± 48.1	0.264	75.7 ± 51.5	75.8 ± 47.8	0.995
**BP parameters at successful recanalization**						
SBP_mean_	119.5 ± 17.6	114.2 ± 12.2	0.112	127.5 ± 15.6	113.8 ± 12.4	0.001
SBP_max_	126.7 ± 70.9	118.7 ± 14.3	0.046	137.5 ± 15.5	118.3 ± 14.7	<0.001
SBP_min_	111.8 ± 17.5	109.8 ± 12.2	0.548	116.7 ± 19.9	109.5 ± 12.2	0.099
SBP_range_	14.8 ± 16.2	8.8 ± 10.0	0.037	20.8 ± 17.0	8.7 ± 10.1	0.001
SBP_SD_	9.1 ± 9.7	5.4 ± 6.1	0.037	12.3 ± 9.7	5.4 ± 6.3	0.002
DBP_mean_	68.4 ± 13.8	63.6 ± 9.5	0.067	72.3 ± 16.4	63.6 ± 19.4	0.001
DBP_max_	71.8 ± 14.2	66.6 ± 10.8	0.076	76.9 ± 15.5	66.6 ± 10.8	0.006
DBP_min_	64.7 ± 14.5	60.6 ± 10.8	0.114	67.4 ± 18.6	60.7 ± 8.9	0.046
DBP_range_	7.1 ± 7.6	5.9 ± 6.2	0.474	9.6 ± 8.5	5.8 ± 6.1	0.077
DBP_SD_	4.3 ± 4.0	3.6 ± 3.7	0.493	5.4 ± 4.1	3.6 ± 3.6	0.137
**Outcome parameters**						
sICH, *n* (%)	13 (72.2)	6 (5.7)	<0.001	8 (80.0)	11 (9.6)	<0.001
SAH, *n* (%)	6 (33.3)	5 (4.7)	<0.001	6 (60.0)	5 (4.4)	<0.001
24 hours NIHSS	22 (15–36)	10.5 (3–17)	<0.001	25 (15–36)	11 (4–19)	0.005
3 months mRS ≤ 2	0 (0)	49 (46.2)	<0.001	0 (0)	45 (43)	0.006

*DBP, diastolic blood pressure; NIHSS, National Institutes of Health Stroke Scale; PH, parenchymal hemorrhage; SBP, systolic blood pressure; TIA, transient ischemic attack; TOAST, the Trial of Org 10172 in Acute Stroke Treatment (TOAST); SAH, subarachnoid hemorrhage; sICH, symptomatic intracranial hemorrhage*.

**Table 2:** Binary logistic regression analyses of associations between blood pressure parameters (per 10 mm Hg increase) and PH.

	**PH**			**PH-2**		
	**OR**	**95% CI**	***P*-value**	**OR**	**95% CI**	***P*-value**
SBP_mean_	1.261	0.861–1.847	0.234	**1.973**	**1.190–3.271**	**0.008**
SBP_max_	1.303	0.953–1.780	0.097	**1.838**	**1.199–2.815**	**0.005**
SBP_min_	1.034	0.689–1.552	0.871	1.443	0.871–2.389	0.154
**SBP**_**range**_	**1.559**	**1.027–2.365**	**0.037**	**1.908**	**1.161–3.136**	**0.011**
**SBP**_**SD**_	**1.998**	**1.017–3.925**	**0.045**	**2.573**	**1.170–5.657**	**0.019**
DBP_mean_	1.261	0.773–2.058	0.354	1.659	0.913–3.014	0.097
DBP_max_	1.256	0.804–1.962	0.317	1.720	0.990–2.986	0.054
DBP_min_	1.172	0.715–1.923	0.529	1.397	0.770–2.538	0.272
DBP_range_	1.393	0.637–3.048	0.406	2.289	0.924–5.669	0.074
DBP_SD_	1.605	0.434–5.940	0.479	3.098	0.681–14.100	0.144

Bold type indicates statistical significance.

DBP, diastolic blood pressure; SBP, systolic blood pressure.

*The associations of each BP parameter with PH were determined using binary logistic regression models adjusted for baseline NIHSS, baseline ASPECTS, and number of device passes, bridging thrombolysis and procedure duration. The associations of each BP parameter with PH-2 were determined using binary logistic regression models adjusted for baseline NIHSS, bridging thrombolysis, and procedure duration*.

Patients with PH-2 had a higher baseline NIHSS score (18 vs. 14, *P* = 0.005), higher 24-h NIHSS (25 vs. 11, *P* = 0.005), and lower rate of 3 months mRS ≤ 2 (0 vs. 45, *P* = 0.006) than those without PH-2. The SBP_max_ (*P* < 0.001), SBP_mean_ (*P* = 0.001), SBP_range_ (*P* = 0.001), SBP_SD_ (*P* = 0.002), DBP_max_ (*P* = 0.006), SBP_mean_ (*P* = 0.001), SBP_min_ (*P* = 0.046) were higher in the PH-2 group. After adjustment for intravenous thrombolysis, procedure duration of mechanical thrombectomy, and baseline NIHSS, the results showed that every increment of 10 mmHg in SBP_mean_ (OR 1.973; 95% CI 1.190–3.271; *P* = 0.008), SBP_max_ (OR 1.838; 95% CI 1.199–2.815; *P* = 0.005), SBP_range_ (OR 1.908; 95% CI 1.161–3.136; *P* = 0.011) and SBP_SD_ (OR 2.573; 95% CI 1.170–5.657; *P* = 0.019) were independently associated with PH-2 (Table 2).

**Figure 2:**
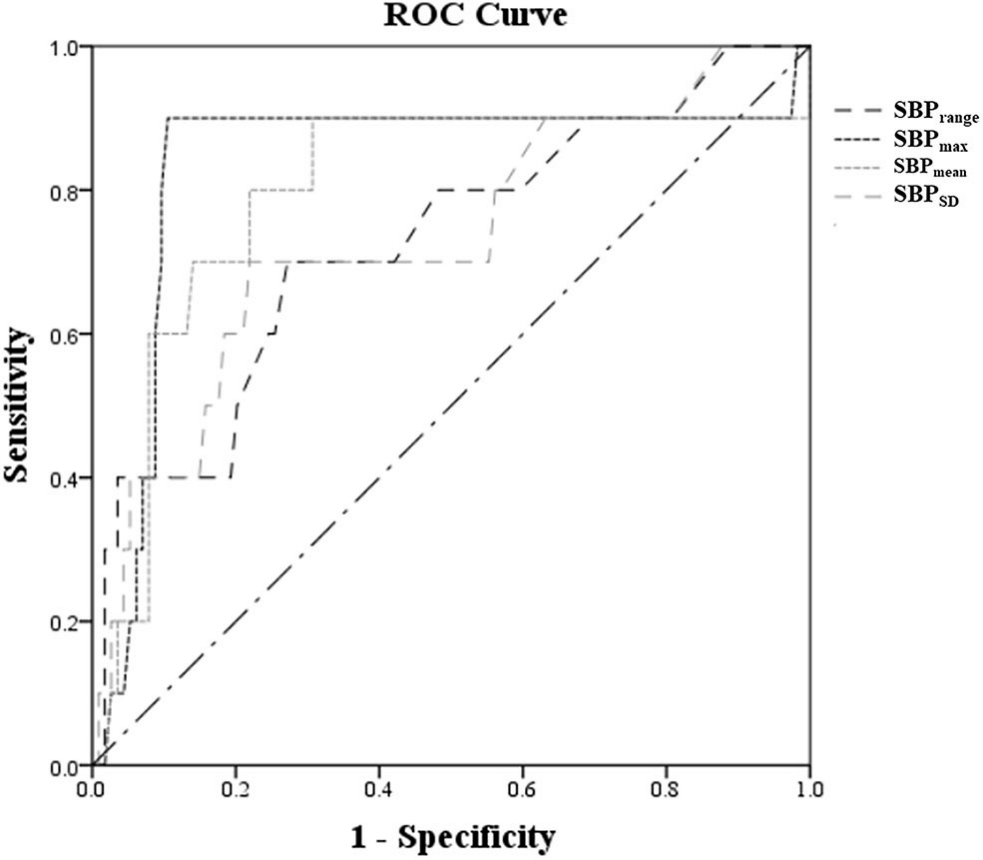
ROC curves of SBP_mean_, SBP_max_, SBP_range_, and SBP_SD_ at the time of reperfusion after IVT to predict PH-2.

**Figure 3:**
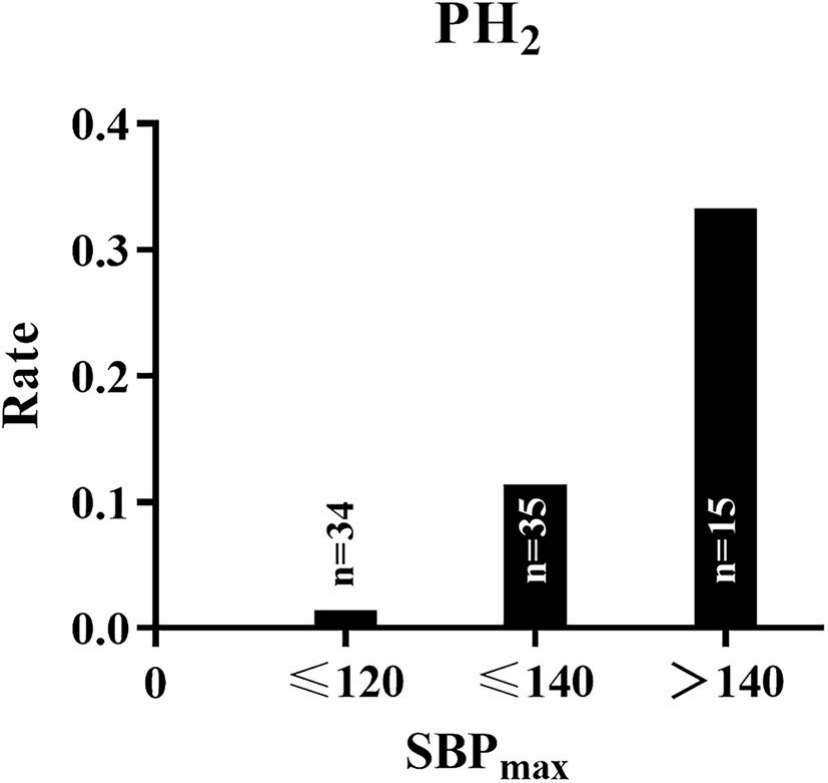
The probability of PH-2 increased with the increase of SBP_max_.

The ROC curves of SBP_mean_, SBP_max_, SBP_range_, and SBP_SD_ in predicting PH-2 are shown in Figure 2, and the areas under the curve (AUCs) were 0.796, 0.836, 0.729, and 0.732, respectively. The optimal cutoffs in predicting PH-2 were 126, 133, 10, and 7.5 mmHg for SBP_mean_, SBP_max_, SBP_range_, and SBP_SD_, respectively (Table 3). Table 3 shows the diagnostic parameters including AUCs, sensitivity, and specificity at the maximal Youden's Index of SBP_mean_, SBP_max_, SBP_range_, and SBP_SD_. As shown in Figure 3, the probability of PH-2 increased with the increase in SBP_max_.

**Table 3:** Predictive value of blood pressure parameters for PH-2.

	**AUC**	**95% CI**	***P*-value**	**Cutoff value**	**Sensitivity**	**Specificity**
SBP_mean_	0.796	0.619–0.974	0.002	126 mmHg	0.700	0.860
SBP_max_	0.836	0.662–1.000	<0.001	133 mmHg	0.900	0.895
SBP_range_	0.729	0.552–0.905	0.017	10 mmHg	0.700	0.728
SBP_SD_	0.732	0.554–0.910	0.015	7.5 mmHg	0.700	0.781

AUC, area of the curve; DBP, diastolic blood pressure; SBP, systolic blood pressure.

## Discussion

Our data suggest that higher systolic BP, measured by SBP_mean_/SBP_max_, and higher BP variability measured by SBP_range_/SBP_SD_ at the time of successful recanalization were consistently associated with a higher likelihood of PH-2 in LVO patients following MT with general anesthesia.

The recommendations of the American Heart Association/American Stroke Association guidelines for BP control in LVO patients treated with MT indicate that an optimal BP target that simultaneously avoids the risk of hemorrhagic transformation and impairment of cerebral perfusion remains unknown (4). Avoiding hypoperfusion injury in ischemic tissue and hyperperfusion injury in reperfused tissue are both essential for BP management after MT (11), indicating that the BP target following MT should not be too high or too low. Several previous studies had demonstrated a U-shaped relationship between admission SBP and mortality in AIS patients (12–14). Given that the overall recanalization rate of MT is high, it seems less important to maintain high BP levels to avoid hypoperfusion injury in ischemic tissue. For patients with successful recanalization after MT, higher BP levels during the first 24 h after MT was correlated with a higher likelihood of sICH and mortality (15). In patients with hyperdensity on immediate non-contrast CT following MT, which indicates risk of hyperperfusion injury, BP levels during the first 24 h were linearly correlated with PH (11). In the present study, we found that SBP_mean_ and SBP_max_ at the time of successful recanalization after MT with general anesthesia were associated with a higher risk of PH-2. Potential reperfusion injury to ischemic tissue may explain why PH-2 was more common in those patients who had higher SBP after MT. Cerebral perfusion pressure is linearly correlated with BP due to impaired cerebral autoregulation following ischemia (16). The increase of systemic BP may result in hyperperfusion injury in ischemic tissues and aggravate blood-brain barrier damage, causing subsequent hemorrhagic transformation (17).

Our study also found that patients with high BP variability measured by SBP_range_/SBP_SD_ at the time of successful recanalization were prone to have PH-2, which is consistent with other related studies. Previous studies had found that blood pressure variability, reflecting the extent of blood pressure fluctuations, could predict unfavorable outcomes in AIS patients receiving intravenous rt-PA (18). Another study in patients treated by intra-arterial therapies showed that SBP variability within the first 24 h was correlated with poor outcomes at 3 months (7). Increased BP variability may lead to instability of cerebral perfusion due to the impairment in autoregulation. The instability of cerebral perfusion pressure in the setting of restoration of blood flow to ischemic tissues may cause disruption of blood–brain barrier permeability and reperfusion injury, resulting in hemorrhagic complications (17, 19). The results suggest that we should not only pay attention to the absolute value of BP control target after MT, but also the stability of BP.

The major difference between our study and other previous studies is that the observation index we focused on is the BP at the time of successful recanalization. Successful recanalization following MT basically solves the risk of hypoperfusion, avoiding reperfusion injury to reduce hemorrhagic transformation becoming more important. Our study suggests that BP has already affected hemorrhagic transformation at the time of successful recanalization in the angio-suite. It may be reasonable to keep BP to low levels and decrease fluctuations since the time of successful recanalization following MT. Interestingly, in line with a previous study (20), minimal BP after MT did not affect the rate of hemorrhagic transformation in our study, which supports the notion that an aggressive BP target is feasible for patients with successful recanalization following MT. The optimal BP control target varies in different studies, which may be related to different study subjects enrolled. In our study, controlling SBP below 120 seems to be beneficial for patients with successful recanalization.

Limitations include the small sample size of the study and its retrospective design, which might have potential for selection bias. Second, we only recorded BP values every 15 min after successful recanalization. It would be more informative to record blood pressure every 3 min or even every minute. Third, we assessed only the relationship between BP and hemorrhagic transformation in AIS patients with anterior circulation LVO occlusion. Therefore, the conclusion can't be simply extended to patients with posterior circulation LVO. Finally, because of limitations of the observational design, the causal relationship between hemorrhagic transformation and blood pressure cannot be determined and prospective randomized controlled trials are needed to address this problem.

In conclusion, patients with higher systolic BP and variability at the time of successful recanalization were more likely to have PH-2 in LVO patients following MT with general anesthesia. Further research is needed to confirm this relationship and the optimal treatment target of blood pressure control after MT.

## Data Availability Statement

The raw data supporting the conclusions of this article will be made available by the authors, without undue reservation, to any qualified researcher.

## Ethics Statement

The studies involving human participants were reviewed and approved by the human ethics committee of The Sir Run Run Shaw Hospital Affiliated with Zhejiang University approved the protocol of this study. The patients/participants provided their written informed consent to participate in this study.

## Author Contributions

HC and CX conducted the statistical analyses and drafted and revised the manuscript. XJ, YC, XZ, FS, XH, YH, and YJ participated in data acquisition and interpretation. ZC and JZ participated in study concept and design, data interpretation and made a major contribution to revising the manuscript. All authors contributed to the article and approved the submitted version.

## Conflict of Interest

The authors declare that the research was conducted in the absence of any commercial or financial relationships that could be construed as a potential conflict of interest.

## References

[1] Powers WJ Rabinstein AA Ackerson T Adeoye OM Bambakidis NC Becker K. 2018 guidelines for the early management of patients with acute ischemic stroke: a guideline for healthcare professionals from the american heart association/american stroke association. Stroke. (2018) 49:e46–110. 10.1016/j.jvs.2018.04.007 29367334

[2] Goyal M Menon BK van Zwam WH Dippel DW Mitchell PJ Demchuk AM. Endovascular thrombectomy after large-vessel ischaemic stroke: a meta-analysis of individual patient data from five randomised trials. Lancet. (2016) 387:1723–31. 10.1016/S0140-6736(16)00163-X 26898852

[3] van Kranendonk KR Treurniet KM Boers AMM Berkhemer OA van den Berg LA Chalos V. Hemorrhagic transformation is associated with poor functional outcome in patients with acute ischemic stroke due to a large vessel occlusion. J Neurointerv Surg. (2019) 11:464–8. 10.1136/neurintsurg-2018-014141 30297537

[4] Malhotra K Goyal N Katsanos AH Filippatou A Mistry EA Khatri P. Association of blood pressure with outcomes in acute stroke thrombectomy. Hypertension. (2020) 75:730–9. 10.1161/HYPERTENSIONAHA.119.14230 31928111 PMC7233454

[5] Goyal N Tsivgoulis G Pandhi A Dillard K Alsbrook D Chang JJ. Blood pressure levels post mechanical thrombectomy and outcomes in non-recanalized large vessel occlusion patients. J Neurointerv Surg. (2018) 10:925–31. 10.1136/neurintsurg-2017-013581 29326379

[6] Goyal N Tsivgoulis G Pandhi A Chang JJ Dillard K Ishfaq MF. Blood pressure levels post mechanical thrombectomy and outcomes in large vessel occlusion strokes. Neurology. (2017) 89:540–7. 10.1212/WNL.0000000000004184 28687721

[7] Bennett AE Wilder MJ McNally JS Wold JJ Stoddard GJ Majersik JJ. Increased blood pressure variability after endovascular thrombectomy for acute stroke is associated with worse clinical outcome. J Neurointerv Surg. (2018) 10:823–7. 10.1136/neurintsurg-2017-013473 29352059

[8] Kleine JF Wunderlich S Zimmer C Kaesmacher J. Time to redefine success? Tici 3 versus tici 2b recanalization in middle cerebral artery occlusion treated with thrombectomy. J Neurointerv Surg. (2017) 9:117–21. 10.1136/neurintsurg-2015-012218 26888952

[9] Neuberger U Mohlenbruch MA Herweh C Ulfert C Bendszus M Pfaff J. Classification of bleeding events: comparison of ecass III (european cooperative acute stroke study) and the new heidelberg bleeding classification. Stroke. (2017) 48:1983–5. 10.1161/STROKEAHA.117.016735 28455322

[10] Adams HP Jr Bendixen BH Kappelle LJ Biller J Love BB Gordon DL. Classification of subtype of acute ischemic stroke. Definitions for use in a multicenter clinical trial. Toast. Trial of org 10172 in acute stroke treatment. Stroke. (1993) 24:35–41. 10.1161/01.STR.24.1.35 7678184

[11] Ding X Xu C Zhong W Gong X Zhou Y Chen Z. Association of maximal systolic blood pressure with poor outcome in patients with hyperattenuated lesions on immediate ncct after mechanical thrombectomy. Journal Neurointerv Surg. (2020) 12:127–31. 10.1136/neurintsurg-2019-014846 31239327

[12] Castillo J Leira R Garcia MM Serena J Blanco M Davalos A. Blood pressure decrease during the acute phase of ischemic stroke is associated with brain injury and poor stroke outcome. Stroke. (2004) 35:520–6. 10.1161/01.STR.0000109769.22917.B0 14726553

[13] Mulder M Ergezen S Lingsma HF Berkhemer OA Fransen PSS Beumer D Baseline blood pressure effect on the benefit and safety of intra-arterial treatment in mr clean (multicenter randomized clinical trial of endovascular treatment of acute ischemic stroke in the netherlands). Stroke. (2017) 48:1869–76. 10.1161/STROKEAHA.117.017996 28432266

[14] Leonardi-Bee J Bath PM Phillips SJ Sandercock PA Group ISTC. Blood pressure and clinical outcomes in the international stroke trial. Stroke. (2002) 33:1315–20. 10.1161/01.STR.0000014509.11540.66 11988609

[15] Anadani M Orabi MY Alawieh A Goyal N Alexandrov AV Petersen N. Blood pressure and outcome after mechanical thrombectomy with successful revascularization. Stroke. (2019) 50:2448–54. 10.1161/str.50.suppl_1.153 31318633

[16] Delgado-Mederos R Ribo M Rovira A Rubiera M Munuera J Santamarina E. Prognostic significance of blood pressure variability after thrombolysis in acute stroke. Neurology. (2008) 71:552–8. 10.1212/01.wnl.0000318294.36223.69 18550860

[17] Liu K Yan S Zhang S Guo Y Lou M. Systolic blood pressure variability is associated with severe hemorrhagic transformation in the early stage after thrombolysis. Transl Stroke Res. (2016) 7:186–91. 10.1007/s12975-016-0458-6 26892891

[18] Endo K Kario K Koga M Nakagawara J Shiokawa Y Yamagami H. Impact of early blood pressure variability on stroke outcomes after thrombolysis: the samurai rt-pa registry. Stroke. (2013) 44:816–8. 10.1161/STROKEAHA.112.681007 23329210

[19] Zhang T Wang X Wen C Zhou F Gao S Zhang X. Effect of short-term blood pressure variability on functional outcome after intra-arterial treatment in acute stroke patients with large-vessel occlusion. BMC Neurol. (2019) 19:228. 10.1186/s12883-019-1457-5 31558167 PMC6764143

[20] Mistry EA Mistry AM Nakawah MO Khattar NK Fortuny EM Cruz AS. Systolic blood pressure within 24 hours after thrombectomy for acute ischemic stroke correlates with outcome. J Am Heart Assoc. (2017) 6:e006167. 10.1161/JAHA.117.006167 28522673 PMC5524120

